# Optimization of sensitivity and specificity of a biomarker-based blood test (LVOCheck-Opti): A protocol for a multicenter prospective observational study of patients suspected of having a stroke

**DOI:** 10.3389/fneur.2023.1327348

**Published:** 2024-02-02

**Authors:** Maximilian Kaffes, Fulvio Bondi, Frederik Geisler, Ulrike Grittner, Lisa Haacke, Thomas Ihl, Maren Lorenz, Marc S. Schehadat, Eugen Schwabauer, Matthias Wendt, Martina Zuber, Dorothee Kübler-Weller, Irina Lorenz-Meyer, Jean-Charles Sanchez, Joan Montaner, Heinrich J. Audebert, Joachim E. Weber

**Affiliations:** ^1^Department of Neurology, Charité—Universitätsmedizin Berlin, Corporate Member of Freie Universität Berlin and Humboldt-Universität zu Berlin, Berlin, Germany; ^2^Center for Stroke Research Berlin, Charité—Universitätsmedizin Berlin, Corporate Member of Freie Universität Berlin and Humboldt-Universität zu Berlin, Berlin, Germany; ^3^Institute of Biometry and Clinical Epidemiology, Charité—Universitätsmedizin Berlin, Corporate Member of Freie Universität Berlin and Humboldt-Universität zu Berlin, Berlin, Germany; ^4^Department of Neurology, Vivantes Klinikum Neukölln, Berlin, Germany; ^5^Department of Neurology, Unfallkrankenhaus Berlin, Berlin, Germany; ^6^Department of Medicine, Faculty of Medicine, University of Geneva, Geneva, Switzerland; ^7^Institute de Biomedicine of Seville, IBiS/Hospital Universitario Virgen del Rocío/CSIC/University of Seville, Seville, Spain; ^8^Department of Neurology, Hospital Universitario Virgen Macarena, Seville, Spain; ^9^Neurovascular Research Laboratory, Vall d’Hebron Institute of Research (VHIR), Barcelona, Spain; ^10^Berlin Institute of Health at Charité—Universitätsmedizin Berlin, Berlin, Germany; ^11^German Center for Cardiovascular Research (DZHK), Partner Site Berlin, Berlin, Germany

**Keywords:** cerebrovascular disease, stroke, large vessel occlusion, prehospital emergency medicine, emergency medicine, mobile stroke units, biomarkers, stroke mimics

## Abstract

**Introduction:**

Acute ischemic stroke (AIS) is a time-critical medical emergency. For patients with large-vessel occlusions (LVO), mechanical thrombectomy (MT) is the gold-standard treatment. Mobile Stroke Units (MSUs) provide on-site diagnostic capabilities via computed tomography (CT) and have been shown to improve functional outcomes in stroke patients, but are cost-efficient only in urban areas. Blood biomarkers have recently emerged as possible alternative to cerebral imaging for LVO diagnosis. Prehospital LVO diagnosis offers the potential to transport patients directly to centers that have MT treatment available. In this study, we assess the accuracy of combining two biomarkers, HFABP and NT-proBNP, with clinical indicators to detect LVO using ultra-early prehospital blood samples. The study was registered in the German Clinical Trials Register (DRKS-ID: DRKS00030399).

**Methods and analysis:**

We plan a multicenter prospective observational study with 800 patients with suspected stroke enrolled within 24 h of symptom onset. Study participants will be recruited at three sites (MSUs) in Berlin, Germany. Blood-samples will be taken pre-hospitally at the scene and tested for HFABP and NT-proBNP levels. Additional clinical data and information on final diagnosis will be collected and documented in an electronic case report form (eCRF). Sensitivity and specificity of the combination will be calculated through iterative permutation-response calculations.

**Discussion:**

This study aims to evaluate the diagnostic capabilities of a combination of the biomarkers HFABP and NT-proBNP in LVO prediction. In contrast to most other biomarker studies to date, by employing MSUs as study centers, ultra-early levels of biomarkers can be analyzed. Point-of-care LVO detection in suspected stroke could lead to faster treatment in both urban and rural settings and thus improve functional outcomes on a broader scale.

**Clinical trial registration:**

Deutsches Register klinischer Studien https://drks.de/search/de/trial/DRKS00030399, DRKS00030399

## Introduction

Acute ischemic stroke (AIS) is a medical emergency requiring rapid action (“time is brain”). The treatment effects of revascularization procedures such as systemic thrombolysis or mechanical thrombectomy (MT) are extremely time-critical (1, 2). To minimize ischemia-related tissue damage and associated functional impairment as well as the need for care and mortality, treatment must be delivered as quickly as possible. Mobile stroke units (MSUs) are specialized ambulances equipped with a computed tomography (CT) scanner with angiography capabilities and a point-of-care (POC) laboratory. These units provide timely and efficient stroke care to patients by bringing the expertise and resources of the hospital directly to the patient’s location. The use of MSUs has been shown to reduce the alarm-to-treatment time (ATT), increase the number of patients treated in the first hour after symptom onset, and improve functional outcomes compared to regular care (3–10). In Berlin, MSUs have been in operation since 2011 as special vehicles of the Berlin Fire Department (Stroke Emergency Mobiles, STEMO).

For stroke patients with a large vessel occlusion (LVO), mechanical thrombectomy treatment is the gold standard (11). Prehospital LVO diagnosis offers the potential to announce and transport affected patients to hospitals that have endovascular treatment available. By avoiding time-consuming secondary transports and accelerating the initiation of therapy, there is a fundamental opportunity for improved outcomes (12, 13). Within routine care in an ambulance, a reliable diagnosis of an LVO has not been possible so far. In contrast, on board an MSU, LVOs can be safely diagnosed by CT-based angiography.

Although the number of operational MSUs in metropolitan areas is increasing and cost-effective (14–16), maintenance, operational and personnel costs are high and MSU coverage is still very limited on a global scale. Therefore, there is a substantial need to explore new and easy-to-manage ways of accelerating reliable identification of LVO in the field without use of computed tomography.

Blood-based biomarkers have emerged as a promising approach for the early detection and diagnosis of stroke, LVO (17–19). These biomarkers offer several advantages over traditional imaging-based methods, including their minimally invasive nature, simplicity, and cost-effectiveness.

Despite the high number of available biomarkers in stroke diagnosis, only a few have shown a sensitivity of more than 50% for stroke in clinical trials, which largely limits their clinical applicability as stand-alone diagnostic tools so far. One approach to solving this dilemma is to try to combine multiple biomarkers in a biomarker panel to gain more useful information (20).

h-fatty acid binding protein has shown potential in differentiating ischemic stroke from stroke mimics due to its rapid release into circulation following the onset of neurological deficits. This biomarker is released from injured cells and has been associated with cellular damage in the brain, making it a promising candidate for early stroke detection (21). NT-proBNP, on the other hand, is a marker of cardiac stress and is elevated in cases of heart failure. Its levels have been observed to rise in patients with acute ischemic stroke, particularly in those with cardioembolic strokes, which are often associated with large vessel occlusions (LVO) (22). Therefore, the combination of these two biomarkers is hypothesized to enhance the accuracy of LVO detection, especially in a prehospital setting where rapid diagnosis is critical for effective treatment.

The vast majority of studies currently underway examine biomarkers in the context of stroke care either after reaching the emergency department or as treatment progresses. A drawback of this method is that these studies do not include patients who are in the very early phase of suffering from stroke symptoms. Changes in concentration of certain biomarkers that may occur exclusively in an early phase are not identified this way.

With LVOCheck-Opti, we aim to address the unmet medical need of prehospital and CT-independent detection of LVO by evaluating a predefined setup of biomarkers. The results of this study will be used to calibrate a point-of-care device which then be tested in a future, independent study.

## Methods and analysis

### Aim of the study

The LVOCheck-Opti study aims to demonstrate that patients with large vessel occlusion can be identified with at least 93% specificity and 66% sensitivity by measuring two blood biomarkers previously identified in acute stroke patients, H-FABP and NT-proBNP, in conjunction with clinical parameters (21, 22). This is achieved by testing biomarker levels in a very early time window in patients suspected of having a stroke when queried by the dispatch center, regardless of the final diagnosis. The approach taken in the present study to detect LVO is based on data from other previously unpublished studies, for example, Biofast 1 and Biofast 2,^1^ presented at ESOC 2021.^2^

The results will be used to train and optimize the test algorithm used in the LVOCheck POCT (point of care test) device. Subsequently, the device will be evaluated in prehospital use in a study independent of LVOCheck-Opti.

As neuronal cell injury quickly progresses with time after onset, we also expect to find an increase in sensitivity and specificity over time. Most studies examine blood samples drawn at a far later stage after AIS, therefore the dynamic course of biomarker levels in the very early stages of stroke is mostly unknown.

Furthermore, we seek to determine the main diagnosis of false positives. We assume that most patients with a false positive test result suffer from AIS without LVO and not from hemorrhagic stroke or stroke mimic. This would consequently result in a transport and treatment strategy optimized for patients with AIS.

### Hypotheses

#### Primary study hypothesis

As primary study hypothesis, we postulate that the measurement of the two previously identified biomarkers H-FABP and NT-proBNP in patients suspected of having an acute stroke will allow the identification of patients with large vessel occlusion with a specificity of at least 93% and a sensitivity of at least 66%.

#### Secondary study hypotheses

As secondary study hypotheses, we postulate that

- The sensitivity and specificity of the two previously identified biomarkers H-FABP and NT-proBNP in patients with acute stroke is higher the less time has elapsed since the event in the first 24 h after symptom onset.
- The main diagnosis of patients identified as false positives by the biomarker combination is non-LVO ischemic cerebral infarction.

### Study design

LVOCheck-Opti is a multicenter prospective observational study of patients with suspected stroke within 24 h of symptom onset based on evaluation by the dispatch center planned to identify sensitivity and specificity of a predefined setup of biomarkers.

### Study population

#### Patient recruitment

We plan to include 800 patients with suspected stroke based on evaluation by the dispatch center between January 20, 2023 and September 30, 2023 at three MSU sites in Berlin. The MSUs are equipped both technically and in terms of personnel for the prehospital diagnosis and care of stroke patients (10). The initial assessment by the dispatch center is performed via telephone using the standard checklist for stroke symptoms.

#### Inclusion criteria

Participants are eligible for the study if they meet the following inclusion criteria:

- Age ≥ 18 years.
- Suspected stroke based on the initial assessment by the fire department’s dispatch center.
- Symptom onset or last seen well (if time of onset unknown) ≤ 24 h.

#### Exclusion criteria

Participants who meet any of the following criteria are excluded from the study:

- Refusal to participate or withdrawal of written consent for study participation by the patient or legal representative.
- Blood sampling is not possible due to any reason.

#### Information and consent

Informed written consent is obtained after the completion of emergency treatment in the MSU. If patients are not capable of giving consent, the consent of the legal representative is obtained. If this is not possible immediately during the emergency medical intervention, it will take place at a later time by the study team.

#### Premature withdrawal of a patient from the study

Participation in the clinical study is voluntary. Each participant has the right to withdraw from the study at any time at their own request, prematurely and without giving reasons, without any disadvantages concerning their further treatment. If a patient declines to participate in the study, the samples and associated data already obtained will be destroyed. As soon as the blood samples and data have been anonymized during the regular course of the study, specific destruction and deletion is no longer possible.

### Outcomes

The aim of the collaborative project LVOCheck is to develop a cost-effective point-of-care test for rapid identification of patients with LVO. The LVOCheck test is intended to be performed prehospitally in regular ambulances so that patients can be transported directly to the nearest thrombectomy center for treatment. The main objective of the subproject LVOCheck-Opti is to evaluate if the measurement of two previously identified biomarkers, HFABP and NT-proBNP combined with clinical parameters, in patients with suspected acute stroke will allow the identification of patients with large vessel occlusion with high specificity and sensitivity.

#### Primary end point

Sensitivity and specificity of the combination of H-FABP and NT-proBNP to LVO diagnosis.

#### Secondary endpoints

Secondary endpoints are

- sensitivity and specificity of the combination of H-FABP and NT-proBNP paired with clinical parameters for LVO diagnosis at different time points after the index event; and
- proportion of false-positive patients with ischemic cerebral infarction in all false-positive patients.

### Collection and processing of data and samples

#### Blood sampling and processing

As part of the routine clinical care on the STEMO, a blood sample is taken from each patient with suspected stroke. An additional sample with a volume of 8 mL will be taken from the patients in addition to this routine blood collection (no additional venipuncture).

The tubes are pre-packed and pre-labeled with unique alphanumeric codes, consisting of a random number generated by the Charité independent data trust office, the number of the aliquot and the type of the sample (H for Heparin, E for ETDA).

One Ethylenediaminetetraacetic Acid (EDTA)-tube (4 mL, purple lid) and one Heparin-tube (4 mL, green lid; BD Vacutainer®, Becton, Dickinson and Company, Franklin Lakes, New Jersey, United States) are used for blood collection. After blood collection, the tubes are turned upside down 8–10 times to mix the blood with the anticoagulant reagent and further processed at room temperature within the next 30 min. Samples are then centrifuged at 2,000 *g* for 15 min (CompactStar CS4, from avantor™ delivered by vwr™, Radnor, PA, United States, and CD-0506, Phoenix Instrument, Garbsen, Germany). The plasma is pipetted from the collection tubes and divided into four aliquots of 500 μL each in cryotubes while maintaining the color code for each sample type (Plasma EDTA → cryotube with purple lid; Plasma Lithium-Heparin → cryotube with green lid). The tubes are then placed inside a special storage box with dedicated compartments for each sample.

The storage box is put into a styrofoam box, stored in the MSU fridge at 4°C until it is transferred to the −20°C Freezer at the respective Fire Department of the MSU site within the next 0.5–4 h. Within 4 weeks, the samples are transported to the biobank at Charité—Campus Benjamin Franklin, where the samples are stored at −80°C. The samples are then sent in batches to the UNIGE team in Geneva on dry ice, maintaining the cold chain. Details of the position of the samples in the boxes and the position of the boxes in the freezer are logged. MSU clinicians will be blinded to biomarker results.

Study-related use of the blood plasma will only commence after the patient or their legal representative has given written informed consent. If no written consent has been obtained in the 12 weeks after sample collection, the patient automatically qualifies as drop out and all samples as well as associated data are destroyed.

#### Description of the laboratory tests

Laboratory testing is performed after transport to the collaborating Translational Biomarker Group, University of Geneva, Switzerland. The analysis focuses on the quantification of HFABP and NT-proBNP levels. Different immunoassays will be used to perform such measurements. These comprise commercial multiplex assays (H-FABP and NTproBNP R-Plex from Meso Scale Diagnostics, MSD), as well as a duplex Point-of-Care test (LVOCheck from ABCDx SA) developed within this project. Furthermore, samples can additionally be used to validate new POCTs that may be developed within this project or tested for additional biomarkers not yet defined at time of sampling.

The data obtained from both biomarkers, together with clinical parameters recorded from the subjects enrolled in the study, will be used to train and enhance an algorithm designed to predict the presence of LVO in subjects with stroke symptoms.

### Documentation of clinical data

Collection of additional clinical data is obtained from MSU treatment documentation (including clinical neurological deficits and NIHSS) as well as by requesting the treatment documentation and doctors’ letters from the hospitals providing information on further treatment and final diagnosis and is documented after pseudonymization.

### Data storage and management

All collected clinical data of the patients are pseudonymized by assigning a unique identification number. This pseudonym is assigned by the Charité data trust office (CHA-THS). Each study site maintains a pseudonymization list in which the patient identification numbers are linked to the full names and date of birth of the participants. This list serves the purpose of later identification of participants. It will be kept confidential and must not leave the study center. It will be archived for at least 10 years after the end of the study. In addition, participation in the study is noted in the patient file. Pseudonomyzed study data are collected in an electronic case report form (eCRF) and managed using Research Electronic Data Capture (REDCap) electronic data capture tools hosted at Charité—Universitätsmedizin Berlin (23, 24).

### Definitions

#### Ischemic stroke

An episode of neurological dysfunction caused by focal cerebral, spinal, or retinal infarction, while infarction is brain, spinal cord, or retinal cell death attributable to ischemia, based on pathological imaging or clinical evidence of cerebral, spinal cord, or retinal focal ischemic injury based on symptoms persisting ≥24 h or until death, and other etiologies excluded (25).

#### Large-vessel occlusion

Occlusion of blood vessels supplying the brain, detectable by angiography.

#### Intracerebral hemorrhage

Rapidly developing clinical signs of neurological dysfunction attributable to a focal collection of blood within the brain parenchyma or ventricular system that is not caused by trauma (25).

#### Stroke mimic

Certain diseases like syncope, epileptic seizure, hypoglycemia, hyperglycemia, migraine aura, dissociative disorder etc. that can cause similar symptoms as stroke. A stroke is initially clinically suspected, but the diagnostic criteria of stroke are not fulfilled (25).

### Statistics

#### Sample size estimation

From internal STEMO data, we know that approximately 14.4% of STEMO patients with stroke alerts have an LVO diagnosis (preliminary study: 260 LVO of 1800 stroke patients). If 800 patients with stroke alarms are included in the study, we can expect about 116 patients to have an LVO diagnosis.

If data from 800 patients are included in the analysis, it is possible to determine a 95% confidence interval for sensitivity ranging from 66.8 to 83.3% if the observed sensitivity is 75%. This confidence interval was calculated using the Clopper-Pearson method and is based on the assumption of a prevalence of LVO of 14.4%. Similarly, with data from 800 patients, it is possible to determine a 95% confidence interval for specificity ranging from 93.3 to 96.7% if the observed specificity is 95%.

Table 1 shows the values for the confidence interval limits in the case of other observed sensitivity and specificity measures.

**Table 1:** 95% confidence intervals for sensitivity and specificity assuming a prevalence of LVO of 14.4% and including data from 800 patients.

Sensitivity	95% confidence interval
65%	56.0–74.1%
70%	61.3–78.7%
75%	66.8–83.3%
80%	72.3–87.7%
Specificity	95% confidence interval
80%	77.0–83.0%
85%	82.3–87.8%
90%	87.7–92.3%
95%	93.3–96.7%

Sample size estimations were performed using nQuery Advisor 8.0 [nQuery (2017). Sample Size and Power Calculation. “Statsols” (Statistical Solutions Ltd), Cork, Ireland].

#### Statistical analysis

A detailed statistical analysis plan will be provided before the analyses of the data.

Descriptive statistical measures such as mean and standard deviation and median and inter quartile ranges will be reported.

##### Statistical analysis of the primary hypothesis

The definitive diagnosis for LVO (gold standard) results retrospectively from the information in the doctor’s letter. Multiple binary logistic regression is used for the algorithm to be developed to predict the individual probability of LVO. Dependent variable: definitive LVO diagnosis from the doctor’s letter, independent variables: Biomarkers H-FABP and NT-proBNP, age, gender, and other clinical parameters and symptoms that can be easily and quickly recorded. The primary outcome analysis yields to evaluate the additional diagnostic value of the biomarkers to clinical and patient characteristics. Since this an observational study, relevant characteristics, that are linked to the outcome will be incorporated in the model for the primary analysis. Using multiple regression models, it is possible to evaluate the additional impact of biomarkers on the outcome after including other patient charactertistics. The method for panel selection will largely follow the procedure outlined by Robin et al. (26), employing panelomics. In summary, we will determine the ideal cutoff values through iterative permutation-response calculations utilizing all relevant parameters (26). The cutoff value will be adjusted in 2% quantile increments, with the sensitivity assessed after each change. This process will continue until we reach peak sensitivity with a specificity of over 95%.

##### Statistical analyses of the secondary hypotheses

In a subgroup analysis, we will only include patients with known symptom onset. To examine time dependency of test sensitivity and specificity, there will be exploratory comparison of subgroups defined by time from symptom onset to sample collection. Sub group differences in sensitivity and specificity measures will be compared descriptively and by calculating odds ratios and 95% CI.

To evaluate the probability of cerebral infarction within false positives, relative frequencies of patients with cerebral infarction in different diagnostic subgroups and odds ratios and 95% CI will be reported.

## Discussion

In this study, our goal is to evaluate a specific set of biomarkers, H-FABP and NT-proBNP, in tandem with clinical metrics, to determine their sensitivity and specificity in identifying LVO in individuals exhibiting acute stroke symptoms. To our knowledge, no attempt has been made to evaluate the combination of these two biomarkers regarding their capability to answer that question in a large cohort.

Previously, some attempts have been made to identify LVO through blood biomarkers. Gaude et al. (17) retrospectively evaluated a range of blood-derived biomarkers for the detection of LVO in two separate patient cohorts with suspected stroke. Diagnostic performance was evaluated using blood biomarkers in combination with National Institutes of Health Stroke Scale (NIHSS) severity scales. Multivariable analysis showed that D-Dimer (OR 16, 95% CI 5–60; value of *p* < 0.001) and GFAP (OR 0.002, 95% CI 0–0.68; value of *p* < 0.05) formed a potential panel for the detection of an LVO. Biomarker scores were combined with scores from different stroke scales. Combining the biomarkers with FAST-ED resulted in the highest accuracy of 95% (95% CI: 87–99%), with a sensitivity of 91% (95% CI: 72–99%) and a specificity of 96% (95% CI: 90–99%). Diagnostic accuracy was confirmed in an independent cohort, where accuracy was also 95% (95% CI: 87–99%), with a sensitivity of 82% (95% CI: 57–96%) and specificity of 98% (95% CI: 92–100%).

Ramos-Pachón et al. (18) were able to identify early D-Dimer levels as an independent predictor of LVO (odds ratio, 1.59 [1.31–1.92]) and observed that a combination of D-Dimer levels and NIHSS scores had superior specificity and positive predictive value to exclude or detect LVO than NIHSS alone.

Chang et al. (19) also used cardiac biomarkers to predict LVO. They found an association between positive troponin and LVO after adjusting for age, sex, and other risk factors (adjusted OR 1.69 [1.08–2.63], *p* = 0.022) and this association persisted after including atrial fibrillation in the model (adjusted OR 1.60 [1.02–2.53], *p* = 0.043).

A number of studies examining biomarkers in AIS without specifically focusing on LVO have identified our target biomarkers as promising candidates for stroke diagnosis. In a study with about 1,300 patients examined from 2012 to 2013, Bustamante et al. (27) investigated the possibility of differentiating patients with ischemic stroke from patients with cerebral hemorrhage and patients with stroke mimic in the acute phase of the disease using a blood-based tool. Some biomarkers, such as NT-proBNP and endostatin, in combination with other variables, were able to achieve a predictive probability of about 80%, but this was not sufficient for accurate diagnosis.

In a pilot study, Zimmermann-Ivol et al. (28) investigated the sensitivity and specificity of NSE, S100B, and H-FABP in patients with acute stroke, acute myocardial infarction, and a group of control patients. Here, H-FABP was shown to have the best sensitivity (68.2%) and specificity (100%) for comparing acute stroke patients versus the control group. In another study by Park et al. (21), plasma H-FABP was shown to be increased in the acute phase of ischemic stroke; however, the diagnostic accuracy of H-FABP as a sole marker was not considered sufficient to be used in the clinical setting.

In contrast to the studies mentioned above, one major limitation will be minimized with our approach. By collecting the blood samples in the ambulance, ultra-early biomarker levels and dynamics can be detected. While the study protocol allows for patients to be included up to 24 h after symptom onset, most of the patients will present with acute symptoms. On the other hand, as ultra-early biomarker levels have not yet been studied extensively, their significance for possible treatment is still uncertain. To accurately assess the effectiveness of biomarkers in early stroke detection, we agree that a focused analysis on the cohort with a precisely known symptom onset time is essential. This approach will enable us to demonstrate the real advantage of rapid data collection facilitated by MSUs, particularly in differentiating acute ischemic events from other conditions. By comparing these findings with data from patients with an unknown onset time, we can further elucidate the temporal dynamics of biomarker changes and their diagnostic utility across different time windows post-stroke.

This study focuses on two distinct biomarkers for LVO detection, but their role in intracerebral hemorrhage still remains unclear. Additional well-studied biomarkers, such as GFAP, might be considered in future studies to further distinguish LVO from hemorrhage (29).

As patients are included in this study based on their assessment by the dispatch center, a wide variety of conditions mimicking stroke will also be considered. While most of these stroke mimics will be hard to distinguish from genuine stroke based on the clinical examination by the on-site neurologist and therefore are a good target for this study, a number of patients will be included where stroke can easily be ruled out at first glance.

## Conclusion

With this study, we aim to evaluate a set of biomarkers regarding their ability to predict LVO ultra-early in a prehospital setting. Utilizing such biomarkers in a POCT could facilitate early decision-making regarding patient transport and treatment. Currently, no biomarkers have been identified that provide high enough diagnostic accuracy to enable such actions.

## Ethics statement

The studies involving humans were approved by Ethics committee of Charité—Universitätsmedizin Berlin. The studies were conducted in accordance with the local legislation and institutional requirements. Written informed consent for participation in this study was provided by the participants’ legal guardians/next of kin.

## Author contributions

MK: Conceptualization, Data curation, Methodology, Project administration, Writing – original draft, Writing – review & editing. FB: Investigation, Writing – review & editing. FG: Investigation, Writing – review & editing. UG: Formal Analysis, Methodology, Writing – review & editing. LH: Investigation, Writing – review & editing. TI: Conceptualization, Data curation, Project administration, Writing – review & editing. ML: Investigation, Writing – review & editing. MS: Investigation, Writing – review & editing. ES: Investigation, Writing – review & editing. MW: Investigation, Writing – review & editing. MZ: Investigation, Writing – review & editing. DK-W: Investigation, Writing – review & editing. IL-M: Conceptualization, Project administration, Writing – review & editing. J-CS: Conceptualization, Formal Analysis, Methodology, Writing – review & editing. JM: Conceptualization, Writing – review & editing. HA: Conceptualization, Writing – review & editing. JW: Conceptualization, Writing – review & editing, Investigation, Supervision.

## Figures and Tables

**Table:** 

AIS	Acute ischemic stroke
ATT	Alarm-to-treatment time
AUC	Area under the curve
BMBF	Bundesministerium für Bildung und Forschung
CHA-THS	Charité data trust office
CSB	Centrum für Schlaganfallforschung Berlin
CT	Computed tomography
CCT	Cranial computed tomography
CTA	Computed tomography angiography
DD	Differential diagnosis
DLR	Deutsches Zentrum für Luft-und Raumfahrt
eCRF	Electronic case report form
EDTA	Ethylenediaminetetraacetic acid
ELISA	Enzyme-linked immunosorbent assay
ESOC	European Stroke Organization Conference
(H-)FABP	h-Fatty acid binding protein
GFAP	Glial fibrillary acid protein
GCP	Good clinical practice
INR	International normalized ratio
JAMA	Journal of the American Medical Association
LVO	Large vessel occlusion
mRS	Modified Rankin scale
MRI	Magnetic resonance imaging
MSU	Mobile stroke unit
MT	Mechanical thrombectomy
NIHSS	National Institutes of Health Stroke Scale
NT-proBNP	N-terminal prohormone of brain natriuretic peptide
PI	Principal investigator
POC	Point of care
POCT	Point of care test
PRESTO	Prehospital Stroke Organization
PTT	Partial thromboplastin time
REDCAP	Research electronic data capture
ROC	Receiver operating characteristic
STEMO	Stroke Einsatz Mobil
TIA	Transient ischemic attack
TT	Thrombin time

## Footnotes

^1^ http://biofast.technology/en/home/

^2^ https://journals.sagepub.com/doi/full/10.1177/23969873211034932
